# Comparing multifocal with unifocal breast cancer and the relationship with survival: national cohort study

**DOI:** 10.1093/bjs/znag033

**Published:** 2026-04-21

**Authors:** Emma Söderberg, Fredrik Wärnberg, Anna-Karin Wennstig, Greger Nilsson, Hans Garmo, Lars Holmberg, Malin Sund, Charlotta Wadsten

**Affiliations:** Department of Diagnostics and Intervention/Surgery, Umeå University, Umeå, Sweden; Department of Surgery, Sundsvall Hospital, Sundsvall, Sweden; Region Västra Götaland, Department of Surgery, Sahlgrenska University Hospital, Gothenburg, Sweden; Department of Diagnostics and Intervention/Surgery, Umeå University, Umeå, Sweden; Department of Oncology, Sundsvall Hospital, Sundsvall, Sweden; Department of Immunology, Genetics, and Pathology, Uppsala University, Uppsala, Sweden; Department of Oncology, Gävle Hospital, Gävle, Sweden; Department of Oncology, Visby Hospital, Visby, Sweden; Department of Surgical Sciences, Uppsala University, Uppsala, Sweden; Department of Surgical Sciences, Uppsala University, Uppsala, Sweden; Translational Oncology & Urology Research (TOUR), School of Cancer and Pharmaceutical Sciences, King’s College London, London, UK; Department of Diagnostics and Intervention/Surgery, Umeå University, Umeå, Sweden; Department of Surgery, University of Helsinki and Helsinki University Hospital, Helsinki, Finland; Department of Diagnostics and Intervention/Surgery, Umeå University, Umeå, Sweden; Department of Surgery, Sundsvall Hospital, Sundsvall, Sweden

## Abstract

**Background:**

The prognostic relevance of multifocal and multicentric breast cancer remains unclear and current staging systems do not consider focality. The aim of this study was to explore whether women with multifocal breast cancer have less favourable tumour characteristics and worse survival compared with women with unifocal breast cancer.

**Methods:**

Patient and tumour characteristics were obtained from Breast Cancer Database Sweden 3.0, which includes data for all Swedish women diagnosed with invasive breast cancer between 2008 and 2019 and who underwent surgery. Overall and breast cancer-specific survival rates were calculated using the Kaplan–Meier method and multivariable analysis was used to identify independent predictors of survival using the Cox proportional hazard model.

**Results:**

A total of 71 419 women were included in the study: 59 445 (83.2%) had unifocal breast cancer, 7286 (10.2%) had multifocal breast cancer with two invasive foci, and 4688 (6.6%) had multifocal breast cancer with three or more invasive foci. Multifocal breast cancer was associated with higher clinical T and N categories compared with unifocal breast cancer. The median follow-up time was 5.96 (interquartile range 3.078.80) years. The breast cancer-specific 10-year survival rates were 86.1% for women with multifocal breast cancer with three or more foci, 86.5% for women with multifocal breast cancer with two foci, and 88.4% for women with unifocal breast cancer. In a multivariable analysis adjusted for patient and tumour characteristics, the HR for breast cancer-specific death was 1.17 (95% c.i. 1.03 to 1.32) for women with multifocal breast cancer with three or more foci compared with women with unifocal breast cancer. There was no statistically significant difference in overall survival between the three groups.

**Conclusion:**

The present study suggests that focality provides prognostic information that is additional to that provided by traditional tumour characteristics.

## Teaser

Multifocal breast cancer was linked to more advanced tumour features than unifocal disease in a nationwide cohort of >71 000 Swedish women. Although overall survival did not differ, women with three or more invasive tumour foci had slightly worse breast cancer-specific survival, even after adjusting for clinical factors. These findings suggest that tumour multifocality has independent prognostic significance.

## Introduction

Multifocal or multicentric breast cancer refers to cancers with more than one invasive focus in the same breast at the time of diagnosis. In multifocal breast cancer, multiple lesions are found in the same quadrant of the breast, and, in multicentric breast cancer, multiple invasive lesions are found in more than one quadrant^1^. There is controversy regarding the outcomes and optimal treatment of multifocal/multicentric breast cancer, hereafter referred to as multifocal breast cancer, compared with unifocal breast cancer. A meta-analysis from 2014 including 22 trials and nearly 70 000 women showed that multifocality was associated with statistically significant worse survival, but there was substantial inter-study heterogeneity, which prevented the precise determination of the increased risk^1^. In 2022, another meta-analysis of 31 trials and 15 000 individuals did not find multifocality to be an independent predictor of poorer survival^2^.

Although some studies have explored the histopathology of all different foci, the results have not influenced the staging system or led to changes in clinical management. According to the AJCC staging guidelines^3^, only the largest invasive focus should be registered, which means that the total size of all lesions together (referred to as the tumour extent) can be considerably larger in multifocal breast cancer compared with unifocal breast cancer. This may lead to an underestimation of the disease burden. The aim of this study was to investigate the distribution of clinicopathological variables and survival for women with multifocal compared with women with unifocal breast cancer.

## Methods

### Study ethics

The study was performed after ethical approval was obtained from the Swedish Ethical Review Authority (EPM Dnr: 2019–04916) in accordance with the principles of the Declaration of Helsinki. Results are reported according to the STROBE criteria for cohort studies^4^.

### Data collection

Data were prospectively collected using the Swedish National Quality Register for Breast Cancer (NKBC). The register was initiated in 2007 and reached national coverage in 2008. It has since been validated, with a coverage of 99.9%^5^. The register includes information regarding diagnostics, tumour characteristics, and therapy for all patients with primary *in situ* and invasive breast cancer in Sweden.

For research use, Breast Cancer Database Sweden 3.0 (BCBaSe 3.0) was created by linking NKBC data to national demographic and healthcare registers, including information about socioeconomic status and co-morbidity. This study included women with primary invasive breast cancer diagnosed between 2008 and 2019. Men were not included as all had previously undergone mastectomy. Women were excluded if they had distant metastases at the time of diagnosis or if they had non-invasive breast cancer only. Additionally, due to the difficulty in distinguishing true multifocality after neoadjuvant therapy, only women who had primary surgery were included.

### Definition of variables

The information collected comprised patient demographics (age and educational level), co-morbidity, treatment data, and tumour characteristics. The expression of oestrogen receptors (ERs), progesterone receptors (PRs), human epidermal growth factor receptor 2 (HER2), and Ki-67 was evaluated using immunohistochemistry (IHC). ER and PR status was considered positive at ≥10% expression, according to current Swedish guidelines. HER2 was scored using IHC as 0, 1+, 2+, or 3+, and by *in situ* hybridization for an IHC score of 2+. Ki-67 scoring was classified as high or non-high using a cut-off that separated the third of tumours with the highest proliferation from the remaining two-thirds. Tumour size was based on the size of the largest invasive focus, irrespective of the number of foci, according to the manual of the AJCC system^3^. Surrogate tumour subtypes were classified using IHC results. Luminal A was defined as ER positive/PR positive, HER2 negative, Nottingham histological grade (NHG) 1–2, and non-high Ki-67, luminal B was defined as ER positive, HER2 negative, NHG 2–3, high Ki-67, and/or PR negative, HER2 positive/luminal was defined as HER2 positive and ER positive, HER2 positive/non-luminal was defined as HER2 positive and ER negative, and triple negative was defined as ER negative, PR negative, and HER2 negative.

Co-morbidity was classified using diagnoses registered in the Swedish National Patient Register in accordance with the Charlson Co-morbidity Index (CCI) using four levels from CCI 0 (no co-morbidity) to CCI ≥3 severe co-morbidity)^6^. Education was classified as low (≤9 years), intermediate (10–12 years), or high (ç13 years), corresponding to mandatory school, high school, and college/university. Multifocality was defined as the presence of more than one invasive tumour foci in the same breast within the same quadrant or different quadrants. This information was registered in the NKBC as the number of invasive foci recorded in the histopathology report. Patients were categorized into three groups, depending on the number of invasive foci registered. See *Fig. S1*.

### Statistical methods

All analyses were performed using the statistical software R version 4.3.1.

Categorical data are presented as numbers and percentages. The median follow-up time was estimated using the reverse Kaplan–Meier method (inverse censoring)^7^. Kaplan–Meier analysis and the log rank test were used to estimate unadjusted overall and breast cancer-specific survival. Crude and adjusted Cox regression models were used to assess the association between multifocality and breast cancer-specific death. In the multivariable analyses, variables considered potential confounders were selected. To avoid inclusion of covariates on the pathway from exposure to outcome, three separate models were created. Model 1 was adjusted for age, educational level, CCI, and detection mode. Model 2 and model 3 were further adjusted for tumour characteristics and treatment variables respectively. Analysis was performed for the whole cohort and separately for subgroups of surgical treatment (mastectomy *versus* breast-conserving surgery (BCS)). The results are shown as HRs with 95% confidence intervals.

## Results

The study included 71 419 women diagnosed with invasive breast cancer between 2008 and 2019 and who underwent primary surgery: 59 445 (83.2%) had unifocal breast cancer, 7286 (10.2%) had multifocal breast cancer with two invasive foci, and 4688 (6.6%) had multifocal breast cancer with three or more invasive foci. See *Table 1* for the baseline characteristics of the study population, presented according to focality. Women with multifocal breast cancer were slightly younger and had higher educational levels. Compared with unifocal breast cancer, multifocal breast cancer was associated with higher clinical T and N categories, a higher Nottingham histological grade, a higher proportion of lobular tumours, and the luminal B subtype. Multifocal breast cancer was also associated with a higher probability of axillary lymph node metastasis (48.2% of women with multifocal breast cancer with ≥3 foci, 39.0% of women with multifocal breast cancer with 2 foci, and 27.2% of women with unifocal breast cancer).

**Table 1:** Baseline characteristics of the study population, presented according to focality

	Unifocal (*n* = 59 445)	Multifocal, 2 foci (*n* = 7286)	Multifocal, ≥3 foci (*n* = 4688)
**Age (years)**			
≤40	2046 (3.4)	327 (4.5)	352 (7.5)
41–60	20 076 (33.8)	2688 (36.9)	2045 (43.6)
61–80	31 910 (53.7)	3578 (49.1)	1979 (42.2)
>80	5413 (9.1)	693 (9.5)	312 (6.7)
**Educational level**			
Low	13 705 (23.1)	1621 (22.2)	862 (18.4)
Medium	24 878 (41.9)	3019 (41.4)	2022 (43.1)
High	20 257 (34.1)	2582 (35.4)	1751 (37.4)
Unknown	605 (1.0)	64 (1)	53 (1)
**CCI**			
0	46 250 (77.8)	5725 (78.6)	3845 (82.0)
1	6854 (11.5)	806 (11.1)	422 (9.0)
2	3427 (5.8)	407 (5.6)	234 (5.0)
≥3	2914 (4.9)	348 (4.8)	187 (4.0)
**Mode of detection**			
Clinical diagnosis	27 686 (46.6)	3569 (49.0)	2434 (51.9)
Screening	31 594 (53.1)	3690 (50.6)	2240 (47.8)
Missing	165 (0.3)	27 (0)	14 (0)
**Clinical T category**			
cT1	42 193 (71.0)	4756 (65.3)	2766 (59.0)
cT2	15 443 (26.0)	2234 (30.7)	1619 (34.5)
cT3–4	1809 (3.0)	296 (4.0)	303 (6.5)
**Clinical N category**			
cN0	53 999 (91.4)	6319 (87.4)	3896 (83.8)
cN1	5075 (8.6)	908 (12.6)	753 (16.2)
**Histology**			
Ductal	45 950 (86.1)	5175 (81.5)	3021 (73.7)
Lobular	7388 (13.9)	1174 (18.5)	1077 (26.3)
**NHG**			
I	13 088 (22.3)	1124 (15.6)	616 (13.4)
II	29 445 (50.0)	3954 (54.9)	2604 (56.4)
III	16 277 (27.7)	2125 (29.5)	1393 (30.2)
**Subtype**			
Luminal A	23 947 (49.2)	2630 (44.4)	1689 (43.5)
Luminal B	13 198 (27.1)	1899 (32.0)	1310 (33.7)
HER2 positive/luminal	4482 (9.2)	627 (10.6)	404 (10.4)
HER2 positive/non-luminal	1989 (4.1)	323 (5.5)	237 (6.1)
Triple negative	5093 (10.5)	448 (7.6)	247 (6.4)
**Pathological T category**			
pT1	40 035 (67.7)	4644 (64.0)	2753 (59.2)
pT2	16 867 (28.6)	2407 (33.2)	1719 (37.0)
pT3−4	2191 (3.7)	200 (2.8)	178 (3.8)
**Pathological N category**			
pN negative	41 697 (72.8)	4304 (61.0)	2353 (51.8)
pN positive	15 561 (27.2)	2754 (39.0)	2191 (48.2)

Values are *n* (%). CCI, Charlson Co-morbidity Index; NHG, Nottingham histological grade; HER2, human epidermal growth factor receptor 2.

### Surgical and medical oncological treatment

Women with multifocal breast cancer were more likely to undergo mastectomy (60.8% of those with 2 foci and 75.2% of those with ≥3 foci) compared with women with unifocal breast cancer (33.3%) (*Table 2*). Accordingly, these women were less likely to receive adjuvant radiotherapy. A higher proportion of women with multifocal breast cancer underwent re-excision surgery. Furthermore, women with multifocal breast cancer were more likely to receive adjuvant chemotherapy (40.8% of those with 2 foci and 51.1% of those with ≥3 foci) compared with women with unifocal breast cancer (32.8%). A higher proportion of women with multifocal breast cancer also received adjuvant endocrine therapy.

**Table 2:** Surgical and oncological treatment for women with unifocal and multifocal breast cancer

	Unifocal (*n* = 59 445)	Multifocal, 2 foci (*n* = 7286)	Multifocal, ≥3 foci (*n* = 4688)
**Breast surgery**			
BCS	39 640 (66.7)	2856 (39.2)	1162 (24.8)
Mastectomy	19 805 (33.3)	4430 (60.8)	3526 (75.2)
**Re-excision surgery**			
Yes	3 882 (6.5)	1033 (14.2)	1017 (21.7)
No	55 496 (93.5)	6239 (85.8)	3664 (78.3)
**Axillary surgery**			
SNB	43 880 (75.8)	4397 (61.7)	2487 (54.2)
ALND	14 042 (24.2)	2733 (38.3)	2103 (45.8)
**Chemotherapy**			
Yes	19 204 (32.8)	2930 (40.8)	2367 (51.1)
No	39 308 (67.2)	4244 (59.2)	2261 (48.9)
**Radiotherapy**			
Yes	43 053 (73.5)	4521 (63.0)	3166 (68.4)
No	15 504 (26.5)	2660 (37.0)	1464 (31.6)
**Endocrine therapy** *			
Yes	44 407 (86.2)	5969 (92.8)	3911 (94.3)
No	6585 (12.8)	402 (6.2)	193 (4.7)
**Anti-HER2 therapy**†			
Yes	1595 (25.1)	217 (23.2)	114 (17.9)
No	4758 (74.9)	719 (76.8)	522 (82.1)

^*^For ER-positive women. †For HER2-positive women. BCS, breast-conserving surgery; SNB, sentinel node biopsy; ALND, axillary lymph node dissection; HER2, human epidermal growth factor receptor 2; ER, oestrogen receptor.

### Survival analyses

**Fig. 1:**
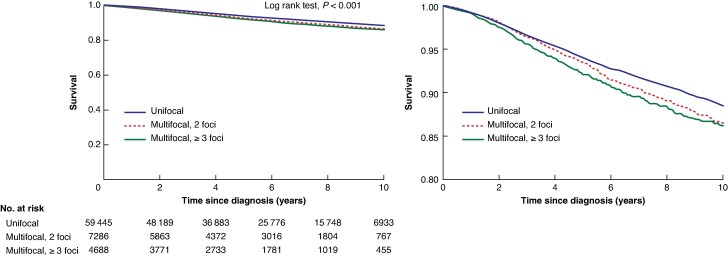
Ten-year breast cancer-specific survival for women with unifocal breast cancer, women with multifocal breast cancer with two foci, and women with multifocal breast cancer with three or more foci

**Fig. 2:**
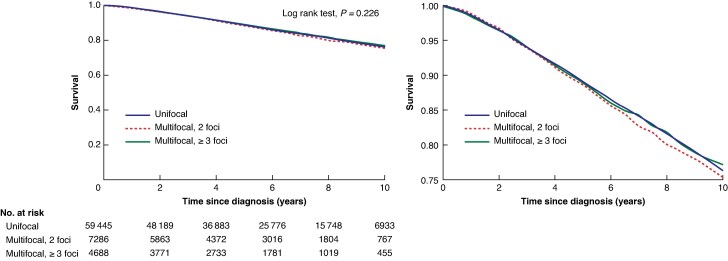
Ten-year overall survival for women with unifocal breast cancer, women with multifocal breast cancer with two foci, and women with multifocal breast cancer with three or more foci

The median follow-up time was 5.96 (interquartile range 3.07–8.80) years. The 10-year unadjusted breast cancer-specific survival was 88.4% for women with unifocal breast cancer, 86.5% for women with multifocal breast cancer with two foci, and 86.1% for women with multifocal breast cancer with three or more foci (*Fig. 1*). There was no statistically significant difference in overall survival between the three groups (10-year overall survival: 76.3% for women with unifocal breast cancer, 75.4% for women with multifocal breast cancer with 2 foci, and 77.1% for women with multifocal breast cancer with ≥3 foci) (*Fig. 2*).

### HRs for breast cancer-specific death

The results of the multivariable Cox regression analyses are shown in *Table 3*. The crude overall HR for breast cancer-specific death for women with multifocal breast cancer with three or more foci compared with women with unifocal breast cancer was 0.86 (95% c.i. 0.76 to 0.97), but, when adjusted for age, educational level, CCI, and detection mode, the HR was 1.08 (95% c.i. 0.96 to 1.22). In the second model, adjusting for tumour characteristics in addition to patient characteristics, multifocality was found to be an independent risk factor for breast cancer-specific death (HR 1.17 (95% c.i. 1.03 to 1.32)). In the third model, adjusting for treatment variables in addition to patient characteristics, an excess risk remained (HR 1.15 (95% c.i. 1.02 to 1.30)).

**Table 3:** HRs of breast cancer-specific death according to surgical treatment and focality adjusted for patient characteristics (age, educational level, CCI, and detection mode), tumour characteristics (pathological T category, pathological N category, morphology, and subtype), and treatment variables (radiotherapy, systemic therapy, and re-excision surgery)

Surgical treatment	Focality	HR (95% c.i.)	Patient characteristics Model 1* HR (95% c.i.	Tumour characteristics Model 2† HR (95% c.i.)	Treatment variables Model 3‡ HR (95% c.i.)
All	Unifocal	1.0 (reference)	1.0 (reference)	1.0 (reference)	1.0 (reference)
	Multifocal, 2 foci	0.80 (0.72,0.89)	0.92 (0.82,1.02)	0.98 (0.89,1.09)	0.96 (0.86,1.07)
	Multifocal, ≥3 foci	0.86 (0.76,0.97)	1.08 (0.96,1.22)	1.17 (1.03,1.32)	1.15 (1.02,1.30)
BCS	Unifocal	1.0 (reference)	1.0 (reference)	1.0 (reference)	1.0 (reference)
	Multifocal, 2 foci	1.16 (0.91,1.48)	1.19 (0.93,1.52)	1.20 (0.94,1.53)	1.18 (0.92,1.51)
	Multifocal, ≥3 foci	1.41 (0.99,2.02)	1.41 (0.99,2.02)	1.27 (0.89,1.82)	1.34 (0.94,1.92)
Mastectomy	Unifocal	1.0 (reference)	1.0 (reference)	1.0 (reference)	1.0 (reference)
	Multifocal, 2 foci	0.75 (0.67,0.84)	0.87 (0.77,0.98)	0.94 (0.84,1.06)	0.90 (0.80,1.01)
	Multifocal, ≥3 foci	0.81 (0.72,0.92)	1.05 (0.93,1.20)	1.14 (1.00,1.30)	1.07 (0.94,1.22)

^*^Adjusted for age, educational level, CCI, and detection mode. †Adjusted for same variables as in model 1 and pathological T category, pathological N category, morphology, and subtype. ‡Adjusted for same variables as in model 1 and radiotherapy, systemic therapy, and re-excision surgery. CCI, Charlson Co-morbidity Index; BCS, breast-conserving surgery.

Separate analyses were also performed with stratification by type of surgery. In these analyses, the HR for breast cancer-specific death for multifocal breast cancer with three or more foci *versus* unifocal breast cancer was higher for women who underwent BCS (adjusted HR 1.41 (95% c.i. 0.99 to 2.02)) compared with women who underwent mastectomy (adjusted HR 1.05 (95% c.i. 0.93 to 1.20)). These results remained consistent in the models adjusting for tumour characteristics and treatment variables.

## Discussion

Women with multifocal breast cancer have less favourable characteristics in general and receive more extensive treatment compared with women with unifocal breast cancer. Multifocal breast cancer appears to be a clinically relevant risk factor for decreased breast cancer-specific survival, with increasing risk as the number of invasive tumour foci increases and for women undergoing BCS. The data from this study indicate that focality can constitute a useful prognostic indicator in addition to the well-established conventional factors included in the current staging system.

In this study of a large population-based cohort, multifocal breast cancer was associated with high-risk features such as larger tumours, unfavourable tumour biology, and axillary lymph node involvement. Accordingly, women with multifocal breast cancer were more likely to receive adjuvant systemic treatments. After adjustment for patient-, tumour-, and treatment-related prognostic variables, multifocal breast cancer with three or more foci was associated with statistically significantly worse breast cancer-specific survival compared with unifocal breast cancer and multifocal breast cancer with two foci. However, for women undergoing mastectomy, no excess risk was observed after full adjustment.

Several studies support the association of multifocal breast cancer with less favourable prognostic features^8,9^. Notably, nearly half of the women with three or more foci had lymph node metastases compared with 27% of women with unifocal tumours. This aligns with Lynch *et al*.^9^, who reported lymph node metastases for 43.1% of multifocal tumours and 27.3% of unifocal tumours. However, there are conflicting results regarding the association between multifocality and survival. The largest study to date included 25 000 women, of whom 6.1% had multifocal disease; similar 10-year overall survival was reported, but worse breast cancer-specific survival was reported, at a relative risk of 1.17 for women with multifocal breast cancer compared with women with unifocal breast cancer^10^. This is also in line with some other studies reporting HRs for breast cancer-specific death ranging between 1.57 and 2.57 for multifocal *versus* unifocal breast cancer^8,11–13^. In a meta-analysis including 22 studies, Vera-Badillo *et al*.^1^ reported that multifocal breast cancer was associated with both decreased overall and breast cancer-specific survival. These findings have been contradicted by others^9,13–16^ and, in a more recent meta-analysis by Zhang *et al*.^2^, multifocality was not independently associated with worse survival. Zhang *et al*.^2^ suggest that this could be explained by advancements in imaging technologies, pathological diagnostic techniques, and therapeutic options. Both of these meta-analyses are hampered by substantial heterogeneity with regard to the included studies and the variability in the definition of multifocality affects the reliability of the results.

To explore whether surgical treatment (BCS *versus* mastectomy) modifies the association between multifocality and survival, a model was constructed, with multivariable analysis stratified by type of surgery. Women who underwent BCS showed a more pronounced association between multifocality and poorer survival. Recently, a study by Yu *et al*.^17^ using Surveillance, Epidemiology, and End Results (SEER) data showed that, for women with multifocal breast cancer, BCS was an adverse factor with regard to breast cancer-specific survival compared with mastectomy. The analysis stratified by type of surgery has limited statistical precision. However, it can be hypothesized that BCS might have been applied to women who had extensive multicentricity, not fully discovered by the preoperative diagnostics. Achieving clear surgical margins is more complex in multifocal breast cancer, which may increase the likelihood of leaving residual disease with metastatic capacity behind. The Alliance study by Boughey *et al*.^18^, which included 204 patients with multifocal breast cancer who had undergone BCS, aimed to study whether it is oncologically safe to perform BCS for multifocal breast cancer and a relatively low rate of local recurrence was reported when negative margins were achieved. In a meta-analysis, multifocal breast cancer was associated with higher rates of local recurrence compared with unifocal breast cancer; however, the differences between the groups decreased when only analysing a subgroup of the more recently included studies, which might reflect the evolution of breast cancer treatment^19^. As the aim of the present study was to analyse survival outcomes, comparisons regarding local recurrences cannot be performed.

As breast cancer survival has improved overall, large sample sizes are required to study differences in survival outcomes. To the best of the authors’ knowledge, this is the largest study assessing survival for multifocal *versus* unifocal breast cancer, with >70 000 women, of whom approximately 11 000 (18.6%) had multifocal breast cancer. The strengths of this study include the large population, with data from validated national registers and with minimal loss to follow-up. Furthermore, the NKBC allows for distinction of multifocal breast cancer based on the number of foci, showing that, as the number of lesions increased, the impact on prognosis became more pronounced.

Some limitations may be considered for the present study. Multifocality or multicentricity is not specifically noted in the register, with only the numbers of invasive foci recorded in the pathology reports being registered. Thus, there is no information with regard to foci being located at particular distances apart or being located in different quadrants of the breast. The locations of tumour foci are generally not consistently reported in the literature, making comparisons between studies difficult. Furthermore, the register does not contain information regarding the tumour biology of the different foci; tumour biology is only registered for one focus, independent of the number of foci found. Therefore, it is not possible to evaluate the importance of heterogeneity, if present, between the different foci.

## Supplementary Material

znag033_Supplementary_Data

## Data Availability

The data are not publicly available due to restrictions according to Swedish and European law, to protect patient privacy. Data are available from the registered holder of BCBaSe 3.0 for researchers with relevant ethical approvals and who meet the criteria for access to confidential data.
