# Supplementary material for: Comparing multifocal with unifocal breast cancer and the relationship with survival: national cohort study
Source: Br J Surg. 2026 Apr 21;113(5):znag033. doi: 10.1093/bjs/znag033 (PMC13150851; doi:10.1093/bjs/znag033)
Supplement: znag033_Supplementary_Data [file znag033_supplementary_data.docx]

omparing multifocality to unifocal breast cancer and the relation to survival: a national cohort study

Emma Söderberg, M.D^1,2^, Fredrik Wärnberg, Prof,^3^, Anna-Karin Wennstig, M.D, Ph.D^2,4^, Greger Nilsson, M.D, Ph.D^5,6,7^, Hans Garmo, Ph.D^8^, Lars Holmberg, Prof.^8,9^, Malin Sund, Prof.^2,10^, Charlotta Wadsten, M.D, Ph.D^1,2^

^1^ Department of Surgery, Sundsvall Hospital, Sundsvall, Sweden

^2^ Department of Diagnostics and Intervention/Surgery, Umeå University, Umeå, Sweden

^3^ Region Västra Götaland, Department of Surgery, Sahlgrenska University Hospital, Gothenburg, Sweden

^4^ Department of Oncology, Sundsvall Hospital, Sundsvall, Sweden

^5^ Department of Immunology, Genetics and Pathology, Uppsala University, Uppsala, Sweden

^6^ Department of Oncology, Gävle Hospital, Gävle, Sweden

^7^ Department of Oncology, Visby Hospital, Visby, Sweden

^8^ Department of Surgical Sciences, Uppsala University, Uppsala, Sweden

^9^ Translational Oncology & Urology Research (TOUR), School of Cancer and Pharmaceutical Sciences, King's College London, London, United Kingdom

^10^ Department of Surgery, University of Helsinki and Helsinki University Hospital, Finland

**Corresponding author**: Emma Söderberg,

Sundsvall Hospital, Department of Surgery

Lasarettsvägen 21

856 43 Sundsvall

Sweden

[emma.soderberg@rvn.se](mailto:emma.soderberg@rvn.se)

**Supplementary Materials - Index**

| **Supplementary Methods** |  |
| --- | --- |
| Detail | *page X* |
| Detail | *page Y* |
| **Supplementary Results** |  |
| Detail | *page X* |
| Detail | *page Y* |
| **Supplementary Appendixes** |  |
| Detail | *page X* |
| Detail | *page Y* |
| **Supplementary Figures and Tables** |  |
| Figure S1: Flowchart of BC included and excluded between 2008 and 2019 | *page 6* |
| Detail | *page Y* |
| **References** | *page Z* |
|  |  |

**Supplementary Methods**

**Supplementary Results**

**Supplementary Appendixes**

**Supplementary Figures and Tables**

**Figure S1:**

**
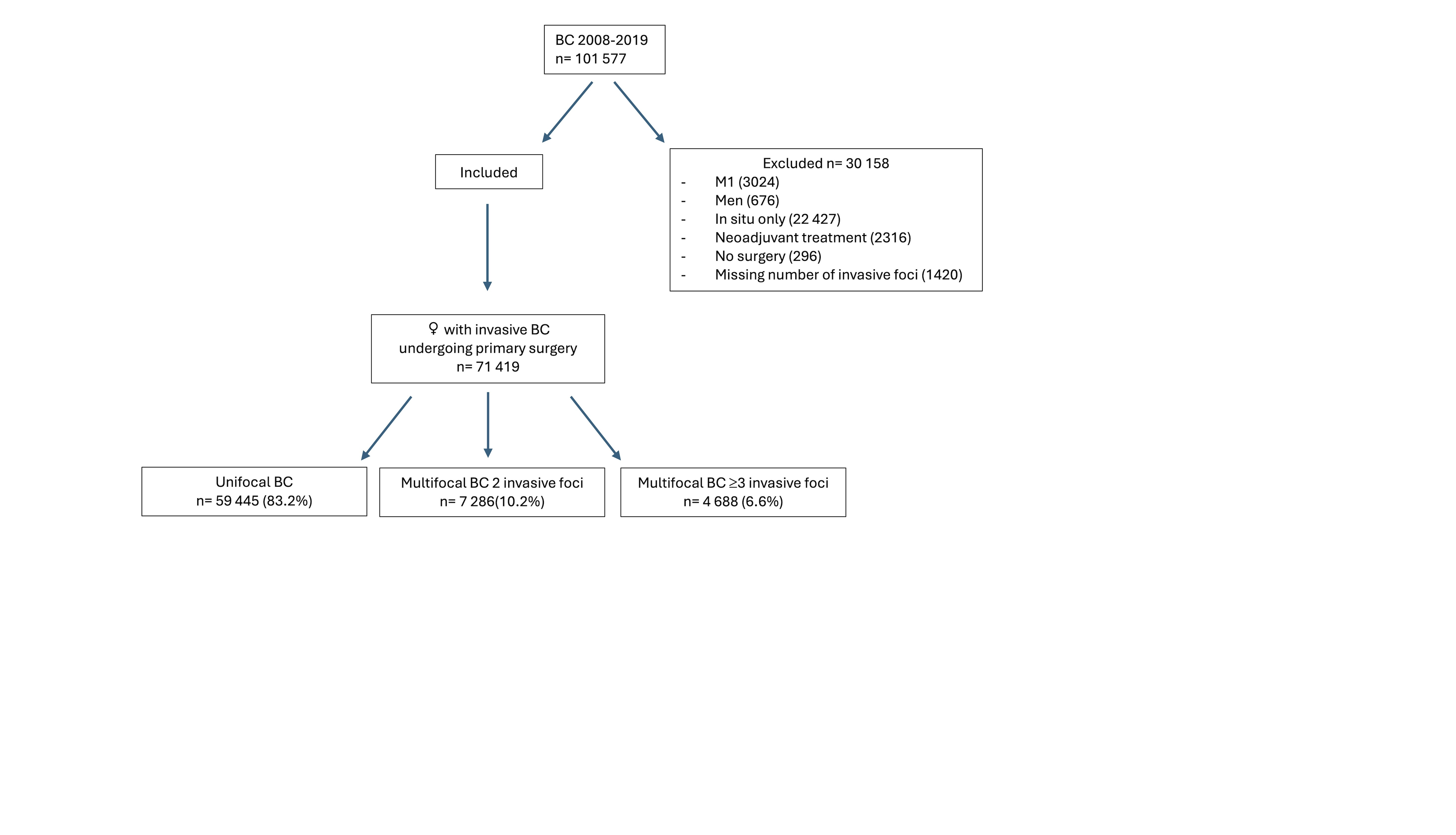
**

**References**
